# Editorial: Outcome assessments for longitudinal studies in pediatric research

**DOI:** 10.3389/fped.2022.884015

**Published:** 2022-09-13

**Authors:** Steven Hirschfeld, Cindy J. Nowinski, Jerry Slotkin

**Affiliations:** ^1^Department of Pediatrics, Uniformed Services University of the Health Sciences, Bethesda, MD, United States; ^2^Department of Medical Social Sciences, Northwestern University Feinberg School of Medicine, Chicago, IL, United States; ^3^Center for Health Assessment Research and Translation, University of Delaware, Newark, DE, United States

**Keywords:** longitudinal study, birth cohort, measurement, pediatric, child health, child development, environmental exposure

## Introduction

The National Academy of Sciences (NAS) report on “Children's Health, the Nation's Wealth” called for new paradigms and techniques for measuring children's health (1, 2).

Health and human development are both multidimensional, multi-layered, and dynamic constructs. Each is benchmarked with a concept of what is considered normal and acceptable, but a wide range of individual circumstances can be compatible with a functional state of health and functional developmental status. While health and development are routinely assessed at a single time, they are understood better when conceptualized and displayed as a quantitative trajectory. The assessment of health is always in the context of development; for example, a healthy status for a 6-month-old infant would be pathological for other ages. While clinical assessments of health are generally directed to detect or evaluate a disease, condition, or other negative influence on health, a research study examining the swath of developmental trajectories across childhood must-have tools and outcomes that capture what is normal and what is exceptional.

## General considerations regarding longitudinal studies

Longitudinal studies are designed and intended to produce informative trajectories. While some longitudinal studies are limited in scope, such as determining the natural history of a particular phenotype or examining the effect of a particular exposure or confluence of exposures on the incidence and severity of a condition of interest, birth cohort longitudinal studies offer the opportunity to gather data on multiple exposures, activities, and outcomes and potentially link these components into influential, dependent, and even causative patterns and sequences.

A longitudinal birth cohort study is generally not intended or designed to provide information or enable decision-making on an individual level, nor to provide feedback in a near time frame to allow responsible health or other outcome decision-making for either an individual or a population. In other words, a longitudinal birth cohort is a long-term knowledge investment and is not suited as a platform for guiding contemporaneous intervention or providing care. These activities must occur in parallel, but independent of the birth cohort study.

Resources, degree of invasiveness, available technology, and time constraints are always limitations on what information can be directly collected, so strategies for acquiring information from multiple sources can augment, integrate with, and extend active protocol-supported data collection, provided there is a framework to integrate and interpret multi-source information.

The scope and types of data to be collected are determined by the framework or the rationale for the study. When a study has a broad mandate to examine exposure-outcome relationships on children's health and development from the environment, where the environment is an all-inclusive term that includes physical, chemical, biological, and psychosocial influences, and to study health disparities as part of the research, it becomes necessary to translate the intent into a program that is scientifically rigorous, feasible, acceptable, and cost-effective.

A comprehensive study with a broad mandate requires acknowledgment of the levels of complexity of assessing and analyzing outcomes, the wide range of outcome options, the knowledge gaps, and the technical and methodological gaps.

The historical lack of a comprehensive system of assessments and age-, gender-, and developmental stage-calibrated measures meant that to comprehensively and successfully address the mandate of a birth cohort study such as the National Children's Study (NCS), the measures and assessment methodologies had to be adapted from prior work or invented and tested and then incorporated into an appropriate framework.

## Specific considerations that guided the NCS

The data collection paradigm the NCS developed in response to the technical need to measure and assess was to focus on objective, precise, and quantitative methods in initial measurements, staying as close to primary physiological properties as feasible, and to use a series of single measurements or clusters of these component measurements to document higher-order or more complex phenotypes. In addition to incorporating and extending the instruments based on Item Response Theory from the NIH Toolbox^®^, the NCS developed an expert working group called the Health Measurement Network to systematically review, evaluate, and propose relevant measurements that were age- and developmentally optimized. Given the combination of multiple, complex goals to achieve and limitations in the assessment methods, the NCS Health Measurement Network systematically evaluated needs and opportunities in multiple domains, producing catalogs of candidate measures and initiating original research to develop new measures. The results of those efforts are summarized in the accompanying papers and include general discussions on motor, sensory, physical health, cognition, social, emotional, and behavioral assessments as well as statistical considerations, incorporating a diverse population and ensuring accessibility for all [Zelazo et al.; Viet et al.; Nowinski et al.; Hirschfeld et al.; Hill et al.; Hays et al.; Harniss et al.; Clark et al.; Hirschfeld; Hirschfeld et al.].

The NCS addressed the complexity through the development of a multidimensional, multi-layered conceptual framework (r6), systematic evaluation and extension of assessment instruments, an organized methodology and workflow for content development, deliberate modeling of individual study visits and the study visit portfolio, and empirical testing of all candidate assessments through formative research and an ongoing pilot study (Hirschfeld et al.).

With the NCS's two-part design, an initial pilot study, known as the Vanguard Study, followed by a larger main study, each part functioning as an operationally independent platform with different, although somewhat overlapping, goals, additional studies could be appended, integrated, or informed by the most relevant platform. Formative research and methods development would fit with the pilot phase and exposure-response relationships would fit with the main study (3).

The NCS data capture plan was resource-intensive, based on the approach of going to the participants rather than having the participants come to a local facility of an existing healthcare delivery system. The rationale was to observe children in the environment in which they live, to psychologically and culturally separate research activities from healthcare delivery activities, and to perform assessments that for the most part are not available at local clinics or even tertiary care centers. Consequently, extensive thought and planning went into the timing, logistics, and resources of each visit to target the overall duration and cost to be within budget. Other studies with a less comprehensive mandate can scale down the schedule and assessments to meet their specific goals.

The Vanguard (pilot) study developed and tested visits only up to the preschool level before the program ended; thus, the comprehensive testing and integration across the full spectrum of childhood remains incomplete. The collected monographs in this series are thus the beginning of an endeavor, with more work to be done.

More details of the proposed linking of the individual age and developmental stage assessments are summarized in the Visit Development manuscript (Hirschfeld et al.), with the interpretation of the assessments into higher-order outcomes implied through the various layers in the conceptual framework. Thus, for the first time, a systematic approach was applied to the challenge issued in the NAS Report to develop outcome measures for longitudinal studies that address the spectrum of both health and development in a conceptual and operational framework.

The degree of investment and expertise required reflected the need to bring rigor and precision to a multidimensional concept of health and to the dynamic and intertwined processes of child development. This collection of monographs is intended to document those efforts and allow others to build on the innovative and comprehensive work of the NCS Health Measurement Network.

## Author contributions

All authors listed have made a substantial, direct, and intellectual contribution to the work and approved it for publication.

## Funding

Life Course Health Sciences (LHS) Working Group was supported by National Children's Study contracts to NORC at the University of Chicago (Contract Number: HHSN275201200004I; Task Order HHSN27500003) and Westat (Contract Number: HHSN275201200005I; Task Order HHSN27500003).

## Conflict of interest

The authors declare that the research was conducted in the absence of any commercial or financial relationships that could be construed as a potential conflict of interest.

## Publisher's note

All claims expressed in this article are solely those of the authors and do not necessarily represent those of their affiliated organizations, or those of the publisher, the editors and the reviewers. Any product that may be evaluated in this article, or claim that may be made by its manufacturer, is not guaranteed or endorsed by the publisher.
